# Root Filling

**Published:** 1894-05

**Authors:** Harry Baldwin


### SELECTIONS.

### Root-Filling.

BY HARRY BALDWIN, M.R.C S., L.D.S., ENG.

Read before the Odontological Society of Great Britain.

The question which arises at the outset, in the consideration of the advisability of immediate root-filling, is as to the nature of the objection which is raised against it.

The objection commonly urged against it, is that pericementitis in some form is more likely to ensue, or become uncontrollable, after immediate root-filling, than after root-filling which has been preceded by previous treatment.

In order, then, to pave the way it seems necessary to state the nature of this pericemental inflammation, and the manner in which its causes come into operation. Everything seems to show that the pericementum primarily becomes inflamed by absorption of some of the products of putrefaction, and some of the micro-organisms which cause putrefaction, both of which previously lodged within the pulp canal; and that it may be kept up by the micro-organisms thus absorbed living and proliferating within the pericementum, and thus supplying fresh quantities of toxic material. For this absorption primarily to take place, it seems to be necessary that these putrid products and organisms should come into actual contact with the pericementum at the apical foramen. Virulently putrid material may be lodged in the pulp-canal without any irritation of the pericementum, provided that it remains entirely within the canal, but if any force intervenes which displaces some of this material out of the end of the root into contact with the pericementum, inoculation may be effected, and inflammation set up. There is nothing to support the idea that the pericementum can ordinarily be infected through the substance of the dentine and cementum; in fact, the channels which unite the dentinal tubules with the canaliculi of the cementum are so exceedingly minute that the tooth substance may here be regarded as a solid wall, effectually barring the progress of irritant matter, excepting in those rare cases where pathologically large channels run transversely between the pulp-canal and pericementum.

It might be objected here that if this theory were all sound, it would be always safe to fill a tooth with putrid pulp-canals at once, without removing the putrid contents, provided no force were used which could displace any of the putrid material into contact with the pericementum. This treatment we know is unsuccessful in practice. The inflammation which follows is probably to be explained, not so much by pressure of malodorous gas upon the pericementum—though the gas would inevitably collect under pressure—but by some of the putrid material being forced into contact with the pericementum by the pressure of this gas. This seems especially likely when we consider that the gas would be generated throughout the mass of the putrid material, and chiefly at the end of the root farthest from the apex, where the putrescent material would be in greater quantity, and the gas from which would be in a better position to project a quantity of the poison against the membrane.

Now, as to the claims of immediate-root-filling.

### ITS ADVANTAGES.

1. It is obviously a great advantage to both patient and operator to be able to immediately permanently fill a root with the certainty of success, in the case of patients who cannot give another sitting.

2. It is obviously a saving of time, as there is but one fixing of rubber-dam, and there is no time lost in placing and removing unnecessary dressings.

3. It is an advantage to plug the apical foramen as soon as safe to do so, as the pericementum is thereby safe-guarded from sundry forms of irritation, not the least of which are sundry pricks inflicted upon it by nerve bristles, and again, the alterations of tension, which the insertion and removal of wet wisps of cotton wool inevitably produce when they fit the channel more or less tightly, as they generally do, and so act as a piston.

4. In addition are the collateral advantages of inspiring a perfection of manipulation and a thoroughness of procedure, which are of great value. It is only by very delicate and thorough treatment, with regard to the removal of putrid material, the avoidance of displacing any through the apical foramen, and the gaining of thorough and direct access as far as possible to all parts of the canal, that success is at all likely to be attained.

ITS DISADVANTAGES.

Provided there is perfect safety in it, there are no disadvantages in immediate-root-filling.

The advantages, however, are considerably lessened by the facts that its perfect safety is in all cases doubtful, unfortunately, and that there is usually an insufficiency of time during a first sitting to carry through all the processes of opening up, cleansing, and filling the cavity of the pulp-canal and the cavity of decay, and again by the facts that a trial dressing takes up but a very little extra time, provided the patient has to come again, and that if followed by serious inflammation, the consequences of immediate-root-fillings are very sad.

It may be noted here that if, as usually happens on a first visit, there is an insufficiency of time to carry through all the processes incidental to the treatment of a dead and putrid tooth, it is advantageous that all the preparation should be done on the first day, and all the filling on the next, if no inflammation of a threatening character has intervened, as by this means the tooth is tested, and very often one thorough drying and application of the rubber-dam suffices, viz., that which has to be done for the fillings.

Now an enumeration of the *dangers* of immediate root-filling.

1. When introducing a nerve-extractor or nerve-canal drill into a root which contains putrid fluid, it is exceedingly likely that the tension so produced will displace some of the putrid fluid into contact with the pericementum, or even quite through the apical foramen. The immediately subsequent attempt at disinfection may fail to neutralize the activity of this poison, and a painful inflammation may be set up, which, if the canal is irremovably filled, cannot be directly treated nor pressure relieved; while the converse is the case if the root-filling admit of easy removal. The danger in this direction would probably be greater in the case of those teeth which were affected by incipient pericementitis at the outset of the treatment, as here the membrane would be of lowered resistance and more prone to become acutely inflamed.

2. When roots are causing abscess without "fistulous" opening there may be a considerable potential cavity in the jaw, eroded by the ulcerative process of the abscess. In many cases it will be impossible to thoroughly sterilize and evacuate this cavity by one operation, and then immediate-root-filling will result in an aggravation of the symptoms, if, indeed, any symptoms previously existed.

Again, supposing that an abscess cavity can be sterilized, but that it remains full of pus when the root is filled, then the best that can happen is that the pus may become inspissated, and represented by a cheesy mass. Mr. Lawson in a clinical lecture, was once very emphatic in declaring that in his opinion not one drop of pus in this world had ever been absorbed, but all cases of seeming absorption of pus were really cases of inspissation. It may be argued that there is no objection to a cheesy mass, but this is not so, as inflammatory changes are more likely to ensue in after-time in the neighborhood of such a dead deposit than at a spot more normally conditioned.

In the opposite method of treatment, viz., the treatment by dressings, the pulp-canal is left open to drain, and antiseptics are introduced at intervals into the canal, and, if possible, pumped through into the potential cavity. Thus the potential cavity becomes more or less rapidly obliterated by the general shrinking together of the inflamed and expanded tissues, if the case be acute, or by the formation of granulation tissue if the case be chronic. In all other parts of the body this is the accredited and most generally successful method of treating abscesses, viz., to combine drainage with disinfection, and so insure the cavity healing up from its base. Thus it is seen that where there is practically an alveolar abscess-cavity in the bone, immediate-root-filling runs counter to all the ordinary acceptations of surgical theory and practice.

Now as to general rules. In every case the canals should be opened up thoroughly and vertically, preferably with German screw-cut burs for the crown, and conical burs for the canals, until instruments can be readily introduced into all the canals without bending.

A good filling for pulp-canals should be solid, unirritating, antiseptic, and removable, and should fill up the whole length of the canal. For reasonably safe roots I prefer as a root-filling a stiff solution in chloroform of gutta-percha and hydronaphthol, plus a sufficiency of cotton-wool fibre to carry it into place, and gutta-percha points where the canals are large. This is easy, penetrating, antiseptic, non-irritating, permanent, solid, and can always be removed with a blunt-pointed nerve-canal drill.

For canals which are not considered very safe ones, or which cannot be followed far owing to curvature of the roots, I recommend a root-filling of a mixture of hydro-napthol, eucalyptus oil, and French chalk on a wisp of cotton-wool. This mixture sets into a crystalline mass, but at the temperature of the body is not too stiff to prevent its being drawn clear away from the canal at will. It is highly antiseptic, and quite unirritating. In a bottle it becomes hard, and has to be melted by heat before it can be used, but when melted remains fluid for a convenient length of time.

### CLASSIFICATION OF ROOTS FOR ROOT FILLING.

1. *Aseptic Roots.*—Those which have never contained putrid material (as where the nerves have recently been killed with arsenic, or have recently died from acute inflammation).

2. *Septic Roots without complications* (*gumboil or abscess; gumboil signifying "sinus"*).

3. *Septic Roots without gumboil but with abscess.*

4. *Sceptic Roots with gumboil.*

1. *Aseptic Roots.*—These should be filled as soon as the nerves are thoroughly out, bleeding ceased, and the canals clean, provided there is no arsenic-irritation. If there be, the canal should be left freely open to the saliva until all tenderness has passed away.

2. *Septic Roots without complications.*—First remove any solid particles of decomposing nerve, or other solid matter from the canal by means of a fine new barbed nerve extractor, introducing it little by little, and meanwhile frequently with-drawing it, and cleaning away from it any engaged particles. This is to prevent, as far as possible, any pumping caused by driving up any piece of solid matter. Then thoroughly dry out with numerous wisps of dry cotton on fine bristles all the fluid contents of the canal. This is emphatically important and should be thoroughly done before any fluid antiseptic is introduced into the canal, as removing the most efficient cause of inflammation. Next, pass a fluid antiseptic, as far as possible, along the canal on a fine wisp of cotton. The antiseptic recommended is mercuric perchloride in alcohol (1 in 1000), or pure carbolic acid. Next, except in the case of unusually large canals, carefully drill out the whole length as far as possible with sterilized probe-pointed Gates' drills, only advancing along the terminal part of the root with a very fine drill, and always avoiding excessively big drills. When using these drills always keep the engine running fast, and press them only gently; never let the engine stop while the drill is in the canal. Now must be decided the question of whether the canals shall be immediately filled. In most cases it would, no doubt, be safe to do so, but in the absence of strong reasons to the contrary, I should put a loose dressing of eucalyptus-oil into the canals, and a temporary cotton and sandarac stopping into the cavity of the crown, reserving the filling of both roots and cavity till another and generally more convenient day, and instructing the patient to take out the temporary stopping if tenderness should occur. On the next occasion, if no serious inflammation be present, and the temporary stopping be still *in situ*, fill the roots and finish the tooth. If the patient were obliged to remove the temporary stopping through tenderness occurring, dress antiseptically again, and renew the temporary stopping, and so continue until the temporary stopping can be retained without discomfort.

3. *Septic Roots without gumboil but with abscess.*—If an abscess be pointing on the gum, open it with a lancet and so convert the case into one with gumboil. When the abscess cannot be opened externally, treatment is often restricted on the first day, with advantage, to merely making a drill-hole or opening into the pulp-canal, and so evacuating pus and relieving tension. When the tenderness is not excessive, open up the canals thoroughly, free them completely from debris, disinfect them well, and if pus is freely forming, leave them open to drain, instructing the patient to merely place cotton dipped in say, camphorated spirit, loose into the cavity during meals, and to take it quite out during the intervals, and to frequently suck the tooth so as to aspirate the pulp-canal.

If the pus is not forming freely, absorb it all as far as possible with dry wisps of cotton or bristles, then well pump an antiseptic (as creasote) along the canal and proceed in the tentative way described above. Do not fill the root till a temporary stopping has been retained tightly in place for, preferably, as long as a week. Do not use gutta percha as a temporary stopping where the patient cannot remove it.

4. *Septic Roots with gumboil.*—A gumboil being a sinus leading from a putrid pulp-canal provides escape for inflammatory exudation, and therefore would prevent any immediate trouble on immediately filling the root, but in order to ensure the healing up of any suppurating cavity which may exist in the jaw, it is better to proceed as follows: Well open up the cavity of the pulp chamber, rake out the contents of the canals, insert into each canal a loose wisp of cotton dipped in creasote or convey creasote into the cavity, if it be in a lower tooth, by means of conveying forceps. If the blades of the forceps be held together and dipped in fluid, they will hold a good quantity of it like a geometric pen, and will drop it when the blades are allowed to separate. Having filled the canals and pulp cavity with the antiseptic, place a round ball of soft unvulcanized rubber in the cavity, then squeeze the rubber in with the largest ended instrument which can be used until the creasote forces out the contents of the sinus and finally issues itself freely through the gumboil. Repeat this process at intervals of several days until the creasote will no longer pass through, penetrating the gumboil with a probe to break open any superficial healing over; then permanently fill the roots.

In very long-standing cases there is a large abscess cavity in the jaw, as proved by probing through the gumboil with a silver probe, the roots may as well be filled immediately. Enlarge the orifice of the gumboil with nitric acid, syringe out the cavity through the hole in the gum with an antiseptic and put in a drainage tube, either of small soft rubber tubing or of gutta percha tissue rolled into the form of a tube. Cut plenty of holes in the sides of the tube. Repeat the syringing at intervals of several days, removing the tube each time and shortening it as the cavity fills up. If it finally refuses to close and the end or ends of the root or roots remain isolated and bare, amputate them with a screw-cut fissure bur.

While the foregoing remarks will be seen to discourage absolute immediate-root-filling, they will be seen to encourage the adoption of all the care and thoroughness of treatment which has been so strenuously advocated by the advanced immediate-root-fillers, and it behooves one to acknowledge fully that their labors and arguments have been of immense service to dental art and science, and though not blindly to be followed in the treatment of every individual case, they have been the means of considerably increasing the general success of root-filling, and shortening the treatment of dead teeth all round. First and foremost among those to whom we are beholden for introducing this new departure into this country, and urging its claims, is George Cunningham, M.A. He has here scored a conspicuous success, viewing the matter broadly.

Before the advent of the immediate root-fillers, dead teeth were approached more or less with fear and trembling, and their treatment was protracted over many weary and unremunerative visits, the very idea of immediate root-filling being looked upon as an absurdity. Now dead teeth have lost most of their terrors and treachery, are approached with a confident feeling, and the number of visits devoted to their treatment may generally be reduced to two.

It is now clear that the main things desirable are to thoroughly and bodily remove all the putrid material from the root without causing inoculation of the pericementum, to disinfect the canal with some potent antiseptic, and to fill it with something permanent and solid; and I think it can be added that in the interest of the patient it is better to wait till the root has had a trial-filling before proceeding to the final stage of the operation.—*British Journal of Dental Science.*

